# An acute coronary syndrome in an unusual patient: analysing cisplatin toxicity—a case report and review of the literature

**DOI:** 10.1093/ehjcr/ytae365

**Published:** 2024-08-02

**Authors:** Josu Erquicia Peralt, Luis Fernandez Gonzalez, Koldobika Garcia San Román, Juan Carlos Astorga Burgo, Aida Acín Labarta

**Affiliations:** Cardiology Department, Cruces University Hospital, Bilbao, Spain; Interventional Cardiology, Cardiology Department, Cruces University Hospital, Bilbao, Spain; Interventional Cardiology, Cardiology Department, Cruces University Hospital, Bilbao, Spain; Interventional Cardiology, Cardiology Department, Cruces University Hospital, Bilbao, Spain; Interventional Cardiology, Cardiology Department, Cruces University Hospital, Bilbao, Spain

**Keywords:** Cisplatin, Germ cell tumour, Acute coronary syndrome, Cardiotoxicity, Case report

## Abstract

**Background:**

Germ cell tumours (GCT) are the most common malignancy affecting young adult men. The introduction of cisplatin-based chemotherapy in recent decades has significantly changed the prognosis of these malignant tumours into highly curable cancer, even in the setting of advanced disease. However, in the last decade, the success of these chemotherapy regimens in curing GCTs has been slowed by a growing recognition of their important late toxicities, such as cardiovascular disease.

**Case summary:**

We present the case of a 23-year-old male, recently diagnosed with a mixed non-seminomatous testicular germinal tumour, on stage IIIA (pT3 cN2 cM1a), with retroperitoneal adenopathies and pulmonary metastases. After performing a right inguinal orchiectomy, he started chemotherapy treatment with cisplatin + etoposide. Shortly after starting treatment, the patient presented an ST-elevation acute coronary syndrome. The cardiac catheterization revealed a non-occlusive thrombus in the middle segment of the right coronary artery. Intracoronary imaging techniques were used to study the arterial wall, which revealed the presence of atherosclerotic plaque that could have ruptured, with the consequent response of platelet aggregation and thrombus formation. Barely 7 months after this event, the patient was again admitted to hospital for pulmonary thromboembolism with pulmonary infarction.

**Discussion:**

To date, there are two hypotheses linking the association between cisplatin-based chemotherapy and cardiovascular disease. The direct hypothesis argues for the presence of direct chemotherapy-induced vascular damage. The indirect hypothesis, on the other hand, is based on the induction and development of cardiovascular risk factors by chemotherapy. This cardiovascular toxicity of chemotherapy is aggravated by a cancer-induced proinflammatory and prothrombotic state.

**Learning points**

- The introduction of cisplatin-based chemotherapy has significantly changed the prognosis of germ cell tumours in young adult men. However, cardiovascular disease has emerged as one of the significant consequences faced by survivors, with a high relative risk of coronary artery disease during the early follow-up.
- There are two hypotheses linking the association between cisplatin-based chemotherapy and cardiovascular disease: direct and indirect hypothesis.
- Cisplatin-induced cardiotoxicity is aggravated by a cancer-induced proinflammatory and prothrombotic state. Consequently, coronary vasospasm and arterial and venous thrombosis are observed on these patients.

## Introduction

Cancer and cardiovascular disease are responsible for half of all deaths in industrialized societies, as well as being the cause of considerable morbidity.^1^ We usually consider these two pathologies to be mutually exclusive, but the increase in life expectancy in both diseases has led to their frequent coexistence in the same patient.^2^ Interestingly, cardiovascular disease is the second leading cause of morbidity and mortality in cancer survivors after recurrent malignancy.

Emerging evidence suggests a relationship between cardiovascular disease and cancer. They share common risk factors (e.g. genetics, age, lifestyle, toxins, and pollution) and might also mutually promote the onset of the respective other disease.^3^ Secondly, chronic inflammation is an indispensable feature of the pathogenesis and progression of both pathologies.^4,5^ Furthermore, during the last two decades, many studies have identified adverse cardiovascular effects of cancer therapies and chemotherapy-induced cardiotoxicity has been deeply researched.

## Summary figure

**Figure:**
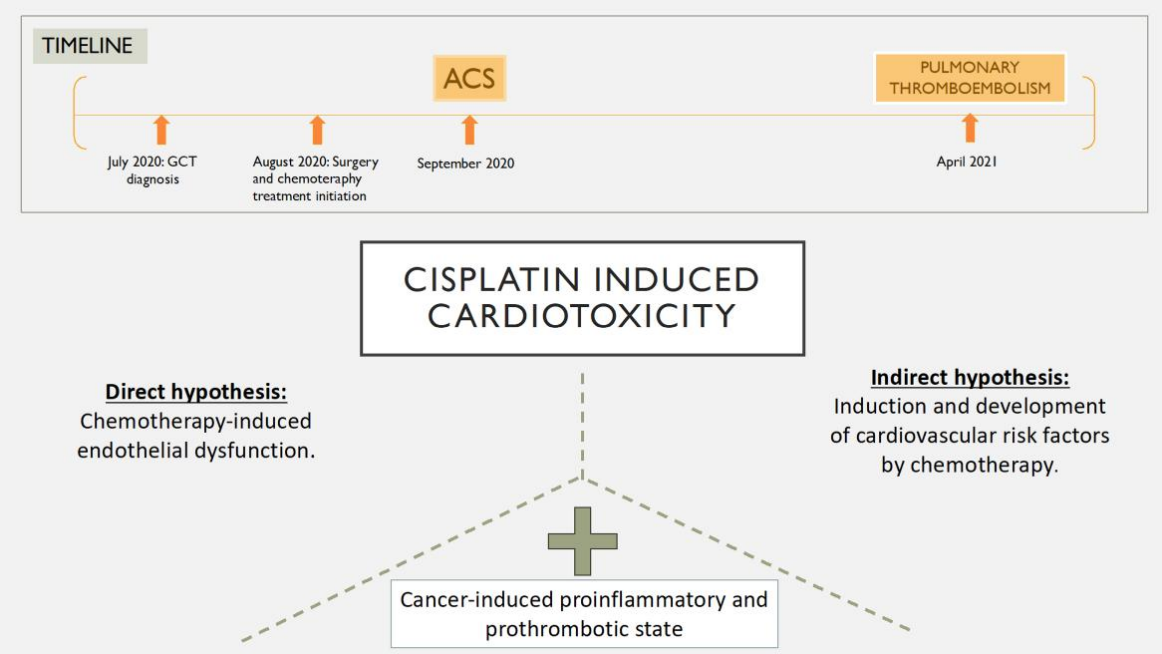


## Case presentation

The patient is a 23-year-old male, a smoker of one pack of cigarettes per day, with no other cardiovascular risk factors of interest and a body mass index of 23 kg/m^2^. He was recently diagnosed with a mixed non-seminomatous testicular germinal tumour, on stage IIIA (pT3 cN2 cM1a), with retroperitoneal adenopathies and pulmonary metastases. After performing a right inguinal orchiectomy, he started chemotherapy treatment with cisplatin + etoposide.

**Figure 1:**
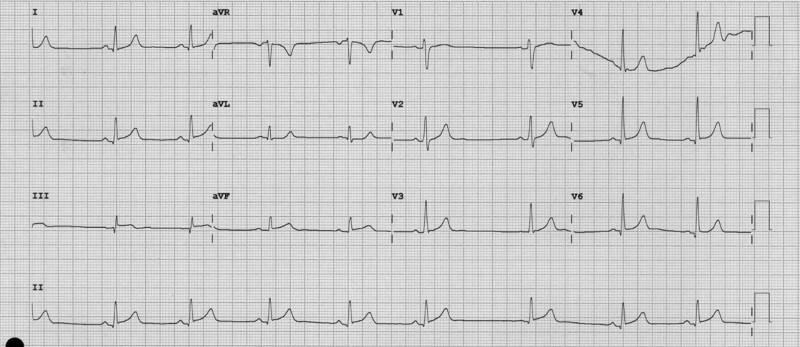
EKG. Sinus rhythm at 55 b.p.m. Axis at 60°. PR is not elongated. ST-segment elevation in leads II, III, aVF, and V5–V6, with specular ST-segment depression in aVL.

A month after starting chemotherapy treatment, at around 4 p.m., he started with oppressive central thoracic pain radiating to the jaw and back, accompanied by persistent cold sweat. He was referred to the emergency department of his reference hospital. On his arrival, haemodynamically stable with blood pressure 129/63 mmHg, in sinus rhythm at 50 b.p.m. but still clinically very affected, an electrocardiogram (EKG) was performed (*Figure 1*). The EKG showed an ST-segment elevation in the inferior and lateral leads. Under the suspicion of an ST-elevation acute coronary syndrome (STE-ACS), cardiac intensive care unit doctors were contacted.

At the arrival of the team, transthoracic echocardiography (TTE) was performed. The TTE showed preserved left ventricular size and function but with hypokinesia of the inferior wall.

Even though the patient was a 23-year-old male, with no cardiovascular risk factors except smoking habit, the referred chest pain and the findings on the EKG, supported by the segmental contractility hypokinesia, oriented the diagnosis to an STE-ACS. Therefore, an emergent cardiac catheterization was performed.

**Figure 2:**
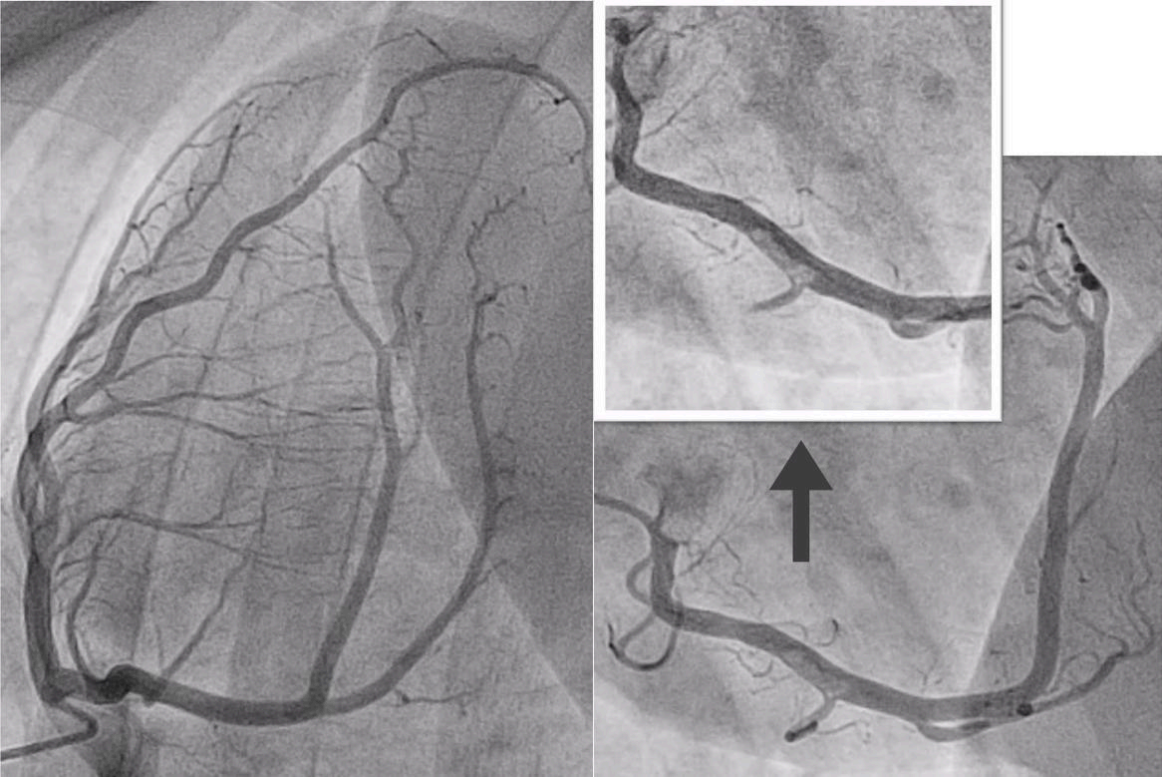
Left and right coronary arteries angiography. Left main coronary artery, anterior descending artery, and circumflex artery with smooth and normal walls. At the mid-segment of the right coronary artery, an image compatible with a thrombus is observed, which does not occlude the artery.

The coronary artery angiography (*Figure 2*) showed the absence of coronary stenoses on the left coronary artery. Nevertheless, at the mid-segment of the right coronary artery (RCA), an image compatible with a thrombus was observed, which did not occlude the artery.

**Figure 3:**
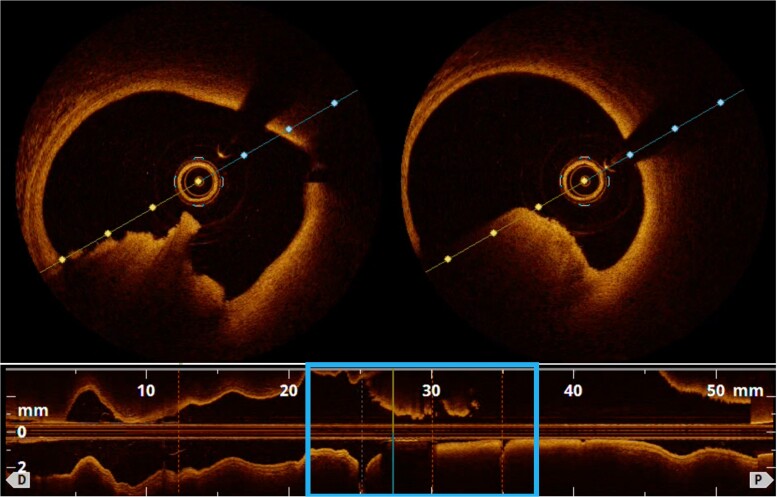
Optical coherence tomography. A mixed thrombus, composed of white thrombus and red thrombus, protrudes into the arterial lumen, with significant light shadowing that does not allow adequate assessment of the arterial wall.

The study was completed with an intracoronary imaging technique to clarify whether the thrombus was associated with complicated atherosclerotic plaque or not. An optical coherence tomography (OCT) was performed (*Figure 3*). The OCT showed an image compatible with a mixed red and white thrombus at the mid-RCA, which produced a light shadow and did not allow an adequate assessment of the arterial wall.

Initially, thromboaspiration was performed on several occasions with a Pronto catheter. However, the thrombus remained adhered to the arterial wall. It was therefore decided to start medical treatment with dual antiplatelet therapy, associated with anticoagulant treatment with low molecular weight heparin.

After 96 h of treatment, new cardiac catheterization was performed. The right coronary artery catheterization showed the persistence of the thrombus at the mid-RCA. A new OCT was performed with similar findings to the previous ones. As a result, after 7 days of admission, the patient was discharged home on treatment with acetylsalicylic acid (ASA), clopidogrel, and anticoagulant treatment with bemiparin.

## Follow-up

**Figure 4:**
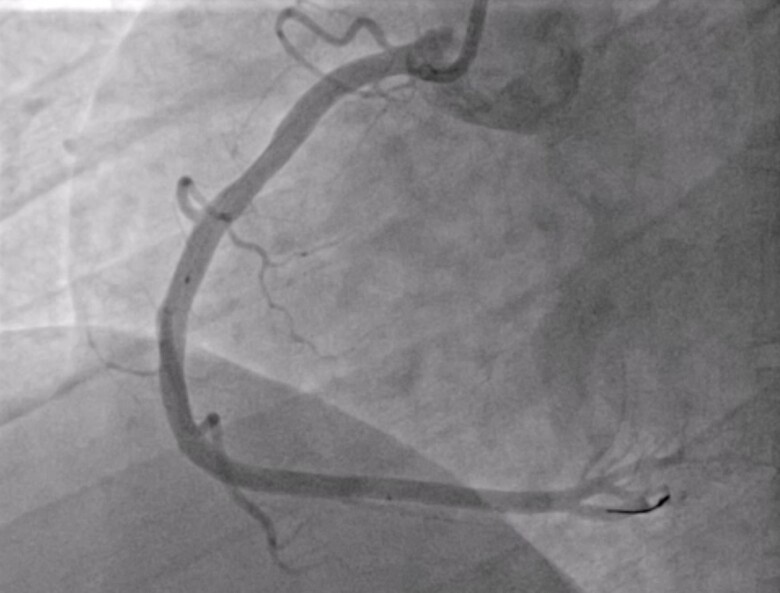
Right coronary artery (RCA) angiography. Slight loss of width in mid-RCA, which could correspond to an atherosclerotic plaque, with no associated thrombotic image.

**Figure 5:**
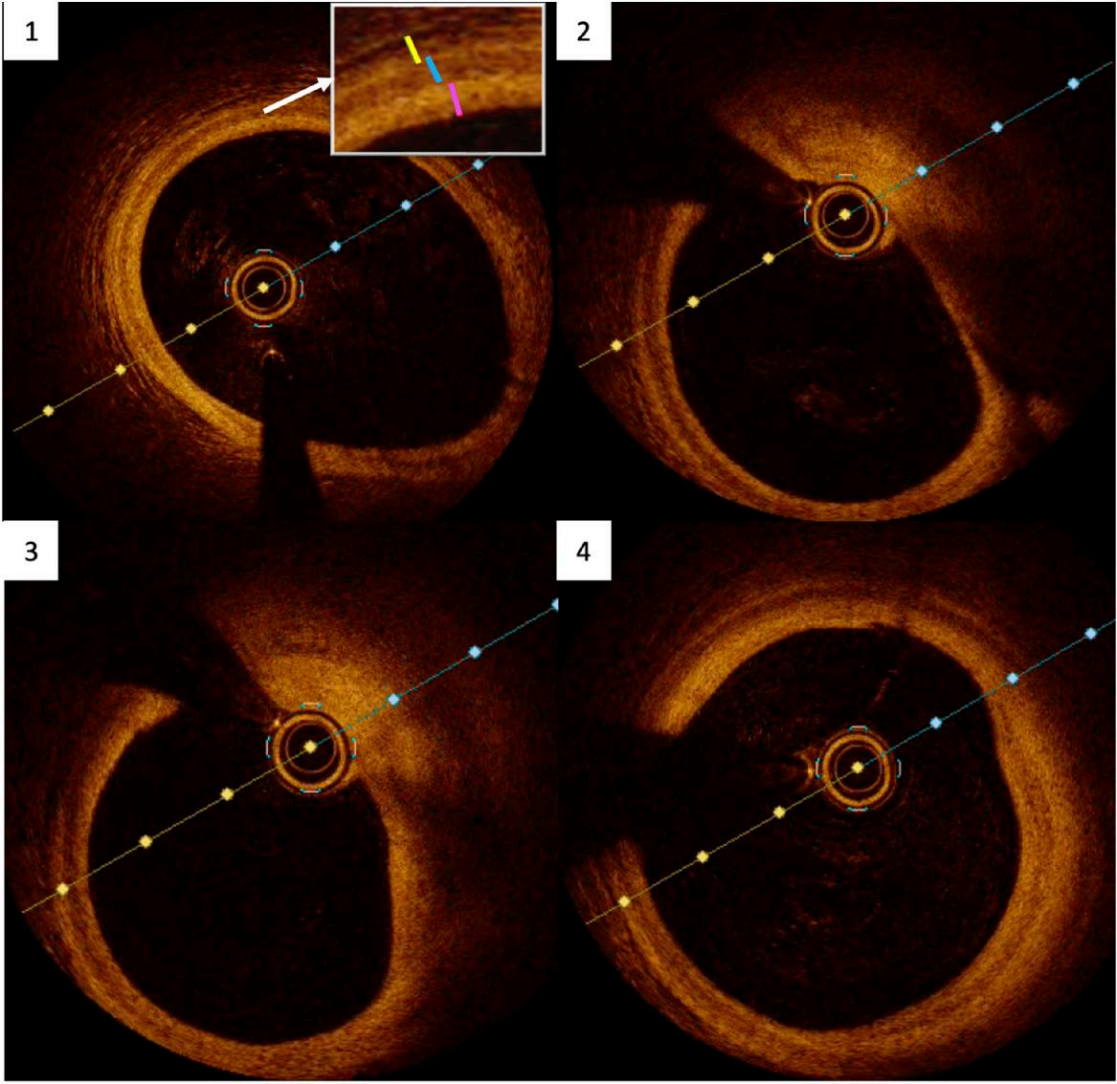
Optical coherence tomography. Panel 1: proximal RCA. The three layers of the artery are visible: tunica intima (red), in contact with the lumen of the artery and showing physiological thickness (<300 μm); tunica media (blue); and tunica adventitia (yellow). Panels 2 and 3: mid-RCA after the first curve of the artery. Between 12 o’clock and 5 o’clock, a pathological thickening of the tunica intima is observed (>600 μm), followed by a thick fibro-lipid layer (>65 μm), which is identified as a heterogeneous mass with lower density than the previous layers and with diffuse borders. Panel 4: mid-RCA before the second curve of the artery. There is a thickening of the intima layer of 500–600 μm, which corresponds to a fibro-lipid plaque.

Four weeks after the ACS, the patient was scheduled for a new cardiac catheterization. On the right coronary artery (*Figure 4*), a slight loss of calibre was observed in the mid-segment of the artery, but with no associated thrombus. A new optical coherence tomography was performed, and all segments of the artery were studied (*Figure 5*).

The OCT showed the presence of stable atherosclerotic plaque in the mid-RCA, without the presence of a thrombus. At the time of this third study, the plaque showed to be stable and did not require percutaneous coronary intervention. However, it could not be ruled out that in the past the plaque could have become complicated, resulting in platelet aggregation with consequent thrombus formation, compromising the flow of the artery and causing the patient’s symptoms.

Based on the findings of the study, two courses of action were taken: anticoagulant treatment was suspended and double antiplatelet therapy with acetylsalicylic acid and clopidogrel were maintained; on the other hand, treatment with a high-potency statin was started.

The patient completed chemotherapy treatment with cisplatin for the following 6 months. At the end of the treatment, a control CT scan was performed that showed complete remission of the tumour. Nevertheless, at the thoracic level, a right upper lobar pulmonary thromboembolism with pulmonary infarction was observed (see Supplementary material online, *Figure S6*).

Given these findings, the patient was admitted to the respiratory ward, and anticoagulant treatment was started. During the admission, an echo-Doppler of the lower limbs was performed, which showed no signs of deep vein thrombosis.

For the following months, the patient received treatment with edoxaban, initially associated with antiplatelet monotherapy. After ensuring the absence of tumour progression and receiving anticoagulant treatment for 6 months, edoxaban was discontinued. By the time anticoagulant treatment was discontinued, one year had passed since the acute coronary syndrome, so maintenance treatment with acetylsalicylic acid 100 mg was established.

Currently, the tumour is still in complete remission and the patient has not presented any new cardiovascular events.

## Discussion

Platinum-containing chemotherapy has brought about a radical change in the prognosis of several malignancies.^6^ However, cardiovascular disease has emerged as one of the significant consequences faced by survivors.^7,8^

The relative risk of coronary artery disease in this population is particularly high during the first 10 years of follow-up when patients are in their 3rd and 4th decades of life.^9^ At present, two hypotheses relate the association between chemotherapy and cardiovascular disease in this population^10^:

### Direct hypothesis: chemotherapy-induced endothelial dysfunction

There is increasing evidence that direct vascular damage underlies the pathophysiology of cardiovascular disease in patients treated with platinum-containing agents. As evidence of this endothelial dysfunction, increased markers of endothelial injury, such as microalbuminuria, and increased levels of platelet adhesion molecules have been documented following exposure to cisplatin compared to patients not exposed to cisplatin.^11^

Furthermore, this cardiovascular toxicity of cancer therapy is worsened by a cancer-induced proinflammatory and prothrombotic state.^8^ Consequently, coronary vasospasm and arterial and venous thrombosis are observed in these patients.^10^

### Indirect hypothesis: induction and development of cardiovascular risk factors

Accelerated development of cardiac risk factors such as dyslipidaemia, arterial hypertension, and obesity has been reported, which partially justifies early atherosclerosis observed in these patients.^10,11^

Metabolic syndrome has been reported in up to 40% of germ cell tumour survivors under the age of 60 years at a median of 11 years post-treatment. It is worth mentioning that there are several studies linking the development of metabolic syndrome to orchiectomy-induced testosterone deficiency. In fact, low testosterone levels have been linked to endothelial dysfunction, vascular hyperreactivity, thickening of the intima and media layer of arteries, and increased synthesis of proinflammatory cytokines.^12^

To summarize, we have presented the case of a 23-year-old patient who, shortly after undergoing a unilateral orchiectomy and starting chemotherapy treatment based on a cisplatin-based regimen, presented two cardiovascular events only 6 months apart.

Concerning ACS, coronary angiography showed an image of coronary thrombosis that was originated from an atherosclerotic plaque that became complicated, producing the consequent response of platelet aggregation. The formation of the plaque could be a consequence of early atherosclerosis (indirect hypothesis). The plaque complication could be related to chemotherapy-induced endothelial dysfunction (direct hypothesis) aggravated by a cancer-induced proinflammatory and prothrombotic state.

On the other hand, pulmonary thromboembolism could be reasoned out similarly. Starting from a cancer-induced prothrombotic state and together with chemotherapy-induced endothelial dysfunction, sufficient to justify the event.

## Conclusion

Cisplatin is a commonly used chemotherapeutic agent for various malignancies. This case highlights the need to be aware of this drug’s cardiac complications, both acute life-threatening ones and those that develop more slowly and could influence the patient 10–20 years after treatment. Therefore, close monitoring of these patients is necessary, initially focused on detecting complications while the patient is being treated with the drug, and subsequently to ensure adequate control of cardiovascular risk factors in the long-term.

## Supplementary Material

ytae365_Supplementary_Data

## Data Availability

The data underlying this article will be shared on request to the corresponding author.

## Lead author biography

Josu Erquicia Peralt (Vitoria-Gasteiz, Spain, 1994) began his medical studies at the University of the Basque Country (UPV) in 2012 and graduated in 2018. He subsequently began his studies in cardiology in May 2019, after completing the entrance exam. In May 2024 he completed his residence in cardiology and currently works as a cardiologist in the cardiology department of Cruces University Hospital.

## Notes

**Consent:** The authors confirm that written consent for the submission and publication of this case report, including images and associated text, has been obtained from the patient in line with COPE guidance.

**Funding:** There was no funding for this work.
