# Supplementary figures and images for: An acute coronary syndrome in an unusual patient: analysing cisplatin toxicity—a case report and review of the literature
Source: Eur Heart J Case Rep. 2024 Aug 2;8(8):ytae365. doi: 10.1093/ehjcr/ytae365 (PMC11310699; doi:10.1093/ehjcr/ytae365)

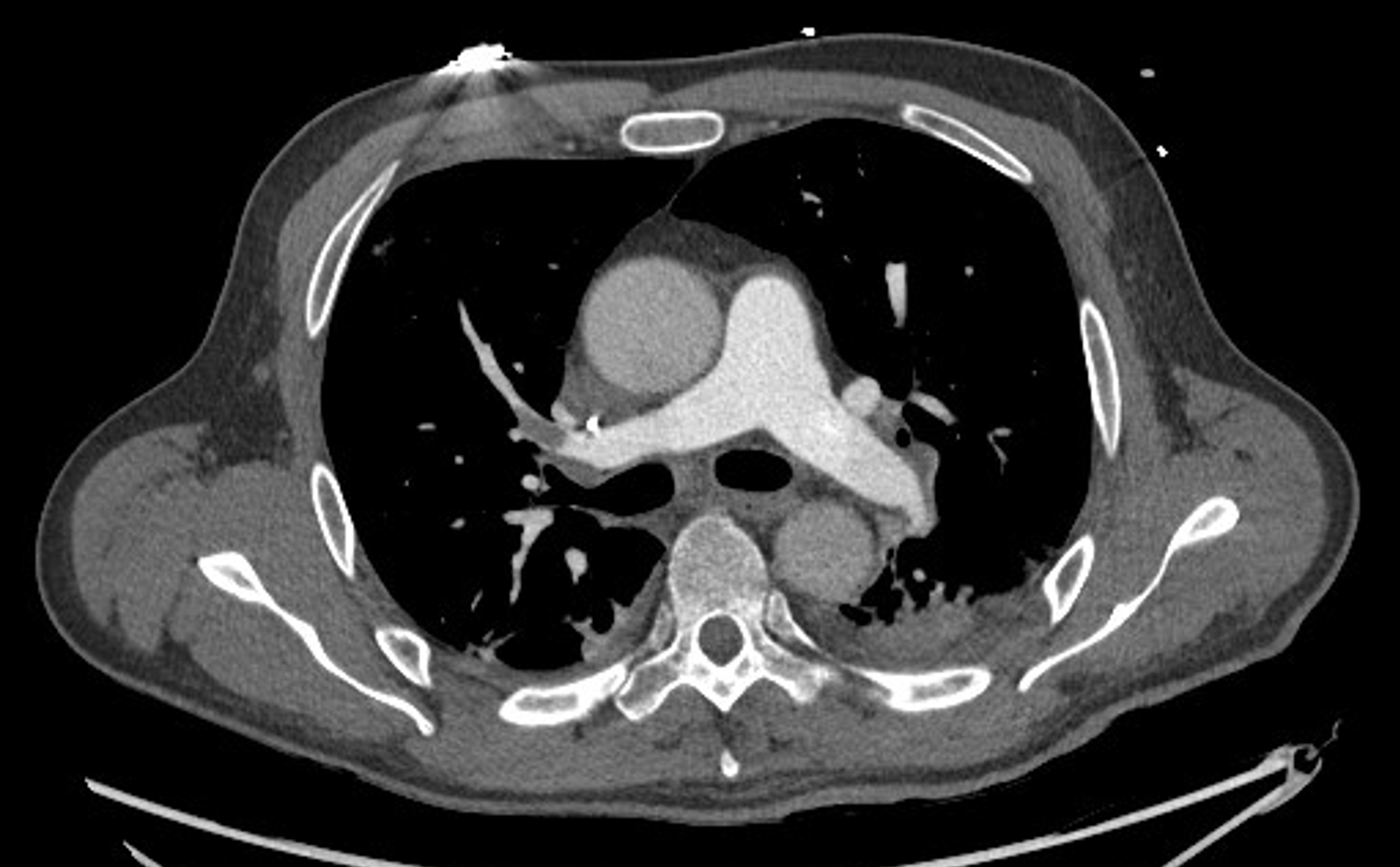

Supplement: ytae365_Supplementary_Data [file ytae365_supplementary_data.zip › FIGURE 6.png]
